# Diversification in Monkeyflowers: An Investigation of the Effects of Elevation and Floral Color in the Genus *Mimulus*

**DOI:** 10.1155/2014/382453

**Published:** 2014-01-05

**Authors:** Ezgi Ogutcen, Brooklyn Hamper, Jana C. Vamosi

**Affiliations:** Department of Biological Sciences, University of Calgary, 2500 University Drive NW, Calgary, AB, Canada T2N 1N4

## Abstract

The vast diversity of floral colours in many flowering plant families, paired with the observation of preferences among pollinators, suggests that floral colour may be involved in the process of speciation in flowering plants. While transitions in floral colour have been examined in numerous genera, we have very little information on the consequences of floral colour transitions to the evolutionary success of a clade. Overlaid upon these patterns is the possibility that certain floral colours are more prevalent in certain environments, with the causes of differential diversification being more directly determined by geographical distribution. Here we examine transition rates to anthocyanin + carotenoid rich (red/orange/fuschia) flowers and examine whether red/orange flowers are associated with differences in speciation and/or extinction rates in *Mimulus*. Because it has been suggested that reddish flowers are more prevalent at high elevation, we also examine the macroevolutionary evidence for this association and determine if there is evidence for differential diversification at high elevations. We find that, while red/orange clades have equivalent speciation rates, the trait state of reddish flowers reverts more rapidly to the nonreddish trait state. Moreover, there is evidence for high speciation rates at high elevation and no evidence for transition rates in floral colour to differ depending on elevation.

## 1. Introduction

The species richness of flowering plant lineages shows tremendous variation amongst clades, indicating that certain traits influence speciation and/or extinction rates. In plants, many traits have been associated with evolutionarily success, such as self-incompatibility [1] and floral asymmetry [2]. The reasons why certain traits are associated with increased diversification are often intuitive: some traits inherently encourage speciation via increased genetic diversity (self-incompatibility) or an association with increased opportunities for the evolution of specialization (floral asymmetry). While geographical distribution might be also influence diversification rates [3, 4] because speciation may accompany the establishment in new ecozones [5], the effects of geographical area are far from fully understood [6].

On the one hand, increased geographical extent provides more opportunities for allopatric speciation (i.e., the “geographical potential for speciation” [7]). If increased geographical extent is simply caused by high dispersal rates, however, gene flow is maintained and hinders speciation [8]. The optimal conditions of dispersal for speciation appear to be met in island systems, where many classic examples of adaptive radiations are found [9]. Recent evidence indicates that island-like systems can also be found in mountainous areas [10], suggesting that mountain peaks also provide the right balance between dispersal and isolation to accelerate speciation, yet these patterns are not as well characterized. One reason why mountain peaks may differ from true island systems is that speciation may occur between lowland and high elevation bands as well as between isolated mountain peaks. In flowering plants, the gene flow between lowland and high elevation bands may decline because pollinators differ along elevation clines, potentially leading to floral isolation [11, 12].

Pollinating fauna exhibit geographical heterogeneity and these differences in composition could play a central role in why angiosperms display such extreme diversity in floral colour and morphology. Lineages of flowering plants can evolve floral colours and shapes that encourage visits from the most efficient pollinator, a process that leads to the observation of “pollination syndromes” [13]. While the absolute nature of pollinator syndromes is debatable and there are numerous examples of exceptions [14], the “bee” functional group is attracted to flowers that are white, yellow, and purple, and birds are noted for their preference for red flowers [4]. Because beetles and flies also often visit white and yellow flowers and infringe on the “bee” portion of the spectrum, the red or reddish hues of the colour palette (including deep oranges, magenta) may be perceived as “catering to a specialized clientele”. The selection pressures that are involved in the evolution of specialization are not currently well characterized nor are the downstream consequences of evolutionary transitions between specialization and generalization. For instance, it has been suggested that hummingbirds are more common at high elevations [15] and this may lead to a speciational pollinator shift accompanied by the transition towards reddish flowers at high elevations.

While floral colour is a noted cue to the most common pollinator of a given species, there are other reasons why the evolution of floral colour is observed to be a labile trait. Research on the genetic architecture of floral colour indicates that drastic colour shifts can occur with a number of single loss-of-function mutations [16]. Macroevolutionary analyses that examine trait conservatism versus node-based shifts in floral colour can shed light on (1) whether any particular floral colour is associated with increased diversification or (2) whether shifts in floral colour are involved in the speciation process itself. Here, we examine these patterns within the genus *Mimulus*, which comprises approximately 120 species [17], mostly restricted to North America [18]. The genus contains a variety of floral morphologies and floral colours [19]. It has been previously shown that there have been multiple shifts from bee pollination to hummingbird pollination within the genus [20]. With ample sequences and robust species-level phylogenies attainable for the genus, the time is ripe for macroevolutionary analysis of diversification patterns in *Mimulus* within a biogeographical context. With a well-resolved phylogenetic tree, *Mimulus* presents an ideal system in which to then test whether (1) transition rates to red/orange/fuschia (“reddish”) flowers are significantly different from the reverse pathway, (2) “reddish” flowers are associated with higher elevations, and (3) “reddish” flowers are associated with differences in speciation and/or extinction rates in *Mimulus*.

## 2. Methods

### 2.1. Dataset

We searched the literature and e-floras (e.g., California Native Plant Society (CNPS, [21]), Calflora [22], Encyclopedia of Life (EOL) [23], and Jepson Herbarium [24]) for qualitative assessments of floral colour of all species of *Mimulus*. Where these were not available, we scanned online pictures and made our own qualitative assessments of colour. In most cases, it was a very straightforward exercise to discern whether the floral colour was in what we considered “reddish” (RO: red, orange, or fuchsia) or not (no-RO), in other words, possessing both carotenoids and anthocyanins. Quantitative assessments of elevation were obtained from e-floras as well, with the admittedly arbitrary cut-off of being restricted to “high elevation” if a species was restricted to sites >1000 m elevation. Similarly, e-floras were also used for assessments of distribution (e.g., Calflora [22], Encyclopedia of Life (EOL) [23], FloraBase [25], United States Department of Agriculture (USDA) - Natural Resources Conservation Service) [26].

### 2.2. Taxon Sampling and Phylogeny Reconstruction

We compiled the available DNA sequence markers for *Mimulus* species on the National Center for Biotechnology Information (NCBI) GenBank website (http://www.ncbi.nlm.nih.gov/genbank/). Our review showed that there are high numbers of species sequenced for 3 markers: (1) *trnL* gene and *trnL-trnF* intergenic spacer (*trnLF*); (2) the internal transcribed spacer region *ITS1*, the 5.8S coding region and *ITS2* (*ITS*); and (3) the external transcribed spacer (*ETS*). The *trnL-F* marker is located in the chloroplast, and *ETS* and *ITS* markers are located in the nucleus. These three markers were used for our phylogenetic analyses, which included 94 *Mimulus* species (Table 2).

*Mohavea breviflora* was used as an outgroup for all analyses, as in previous molecular work [27].

DNA sequences for *ETS*, *ITS*, and *trnL-F* markers were aligned using Mesquite version 2.75 [28] with Opal package [29] using default settings. The same Mesquite package was used to concatenate the DNA markers for each taxon. Analyses were made with combined nuclear markers (*ETS* and *ITS*) and for all markers (*ETS*, *ITS*, and *trnL-F*). For partitioned analysis of the combined data, nexus files were manually edited for partition setting, in which each DNA marker was set as an individual partition. The three DNA markers were analyzed both individually and in combination for phylogenetic reconstruction analyses.

Rapid bootstrap analysis and search for a maximum likelihood (ML) tree were performed with Randomized Axcelerated Maximum Likelihood (RAxML) software version 7.4.2 [30] under the general time reversible (GTR) substitution model with gamma rate heterogeneity for all individual and combined data sets. *Mohavea breviflora* was included as the outgroup prior to analysis. Furthermore, Bayesian analyses were conducted for all data sets using BEAST v1.7.4 [31]. BEAST.xml files for each data set were generated using BEAUti v.1.7.4 [32]. *Mohavea breviflora* was used as an outgroup, and the ingroup was assigned to be monophyletic prior to analysis. Hasegawa, Kishino, and Yano (HKY) model [33] with gamma site heterogeneity was used as the substitution model due to its general reduction of assumptions. We used the node splitting *Mimulus* from the outgroup *Mohavea* as our calibration point for our phylogenetic tree. In order to take calibration uncertainty into account, we used normal distribution as prior to our calibration node with a mean of 76 Ma and standard deviation of 1 [34]. The rest of the node ages were estimated using uncorrelated lognormal relaxed clock model with the mean distribution prior set to gamma. We selected the Yule speciation model [35, 36] with uniform distribution as our tree prior. For the Markov chain Monte Carlo (MCMC) analyses, the chain length was set to 10,000,000 and we logged parameters every 1,000 generations (i.e., at the end of the run, we had 10,000 samples). For runs with combined data sets, separate models were allowed for each partition. Maximum clade credibility (MCC) trees were generated using TreeAnnotator version 1.7.4 (part of the BEAST package) with the first 50 steps discarded at the start of the run.

### 2.3. Phylogenetic Comparative Analysis

#### 2.3.1. Macroevolution of Reddish Flowers

We used the binary-state speciation and extinction (BiSSE) method [37] implemented in the “diversitree” package [38] of the *R* statistical software [39] to examine differences in transition rates between character states as well as differences in speciation rates. We used this method to estimate the following parameters: speciation and extinction rates *λ*_0_ and *μ*_0_ of nonred lineages and *λ*_1_ and *μ*_1_ of red lineages and two transition rates representing the evolution of red flowers (*q*_01_) and the backwards transitions of evolving nonred flowers (*q*_10_), and then compute the likelihood of character states at the tips of our phylogenetic tree, given the maximum likelihood values of speciation, extinction and transition rates (the unconstrained model) [37]. We then constrain certain parameters to be equal to each other and test alternative models of evolution against the unconstrained model with likelihood ratio tests. We examined the fit of a model with (1) equal speciation rates (*λ*_1_ = *λ*_0_), one with equal extinction rates (*μ*_1_ = *μ*_0_), one with equal speciation and extinction rates (*λ*_1_ = *λ*_0_ and *μ*_1_ = *μ*_0_), and, finally, one with equal transition/migration rates (*q*_10_ = *q*_01_).

We further formalize ancestral reconstructions in Mesquite version 2.75 [28] to characterize the evolutionary history of *Mimulus*. We first used unordered maximum parsimony (MP) reconstruction for standard categorical data. For the second analysis, we performed ML reconstruction with Markov *k*-state 1 model for standard categorical data. MCC tree obtained from the Bayesian analysis with combined DNA sequence data is used for both ancestral reconstructions. Red (or reddish) floral colour is denoted as 1, and all the other floral colours are denoted as 0 in the character matrix. Finally, we established whether the origins of floral colour coincided with entry into specific biogeographical origins using Reconstruct Ancestral State in Phylogenies (RASP) version 2.1 beta [40] software. Three alternative reconstruction methods were used: (1) Statistical Dispersal-Vicariance Analysis [41], (2) Bayesian binary method [42], and (3) dispersal-extinction-cladogenesis model [43]. Four discrete states were used for present-day continental geographical distribution: North America, South America, Asia, and Oceania (Table 2). Analyses were conducted on the maximum clade credibility tree generated on BEAST using combined sequence data, and the results were summarized as tree graphs and pie charts.

#### 2.3.2. Diversification at Different Elevations

The GeoSSE functions within “diversitree” can be used to test differential speciation and extinction within different geographical regions as well as the speciation that might accompany range shifts. We implemented GeoSSE within “diversitree” to examine whether clades at high-elevation bands experienced greater speciation. The framework is very similar, but the parameters are speciation within low elevation regions (with range restricted to <1000 m; sA), speciation within high elevation regions (with range restricted to >1000 m, sB), between-region speciation (sAB), extinction from low elevation regions (xA), extinction from high elevation regions (xB), dispersal from A to B (range expansion, dA), and dispersal from B to A (dB). We coded the species in three categories: A = restricted to low elevations, B = restricted to high elevations, and AB = ranges that span low and high elevation. By setting certain constraints we can test for whether speciation, extinction, or range expansion is different between regions. We also examine the variation in the estimates of these rates with Markov models.

#### 2.3.3. Correlations between Red(dish) Flowers and High Elevations

A final analysis, MuSSE, was run to test whether red flowers are correlated with high elevation zones. In the MuSSE analysis, trait combinations use the following notation: 1: low elevation, no-RO; 2: low elevation, RO; 3: high elevation, no-RO; 4: high elevation, RO. We test whether the evolution of the RO phenotype depends on the background of elevation (does 1 → 2 differ from 3 → 4 or 1 → 3 from 2 → 4, etc.) by constraining transition rates (*q*_12_ = *q*_34_, *q*_13_ = *q*_24_, *q*_21_ = *q*_43_, and *q*_31_ = *q*_42_) and comparing the likelihood of the tip character states on our phylogeny with log-likelihood tests.

## 3. Results

### 3.1. Data Set

We provide the compiled traits of *Mimulus* species in Table 2. We were able to obtain high sampling of sequence data for *Mimulus*, covering 94 of the ~120 species (~79% of the species were included in our analysis). As well, we had unbiased covering of the species in *Mimulus*; 10.6% of the species in our phylogeny are RO, identical to the approximately 10.6% in the genus as a whole that we estimate to be RO (see Table 2).

We had two nuclear markers: *ETS* and *ITS* with sequence data for 90 and 91 taxa for these markers, respectively. After the alignment of all the taxa, the total length of the *ETS* and *ITS* regions analyzed was 477 and 688 bp, respectively. For the chloroplast marker *trnL-F*, we had sequence data for 85 taxa. Once all the taxa were aligned, the analyzed *trnL-F* region was 1122 bp long. When we align and combine all three markers (two nuclear and one chloroplast) together, the resulting matrix contained 2287 characters, comprising 94 taxa excluding the outgroup.

### 3.2. *Mimulus* Phylogeny

**Figure 1:**
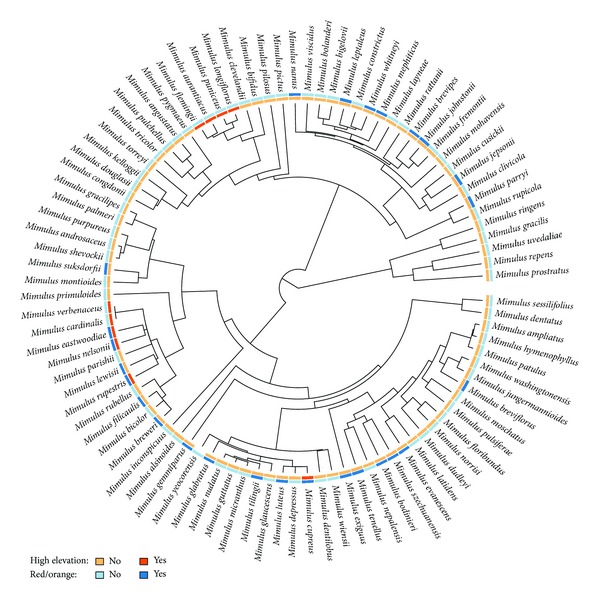
Ultrametric phylogeny of *Mimulus *with red/orange (RO) and restriction to high elevation (blue shading) mapped onto the tips.

ML and Bayesian analyses of the individual DNA markers resulted in largely congruent topologies for the genus *Mimulus* (data not shown). When we compare individual marker data with the combined sequence data, combined molecular analysis provided higher resolution and more consistent tree topologies. We used partitioned and nonpartitioned combined data for ML analyses, and they both provided the same tree topology with slightly different likelihood values (data shown only for nonpartitioned analysis). Therefore we used nonpartitioned ML tree for further analyses. Overall, for the combined DNA sequence data, MCC tree from the Bayesian analysis is largely congruent with the ML analysis (Figure 1), with the Bayesian tree having higher resolution (see Supplementary Figure 1 in the Supplementary Material available online at http://dx.doi.org/10.1155/2014/382453, Supplementary Figure 2). Overall, the phylogeny inferred for *Mimulus* is very similar to the previously published phylogenies using alternative sampling and methods [17–20, 44].

### 3.3. Ancestral Reconstruction

All three biogeographical origin reconstruction methods show strong support (93.83%) for the North American origin for the genus *Mimulus* (see Supplementary Figure 3 for Bayesian Binary MCMC Analysis). Both ML and Bayesian analyses highly supported the clade with Oceanic distribution consisting *M. prostratus*, *M. repens*, *M. uvedaliae*, and *M. gracilis*, with bootstrap value (BS) of 100% and posterior probability (PP) of 1.0. North American taxon; *M. ringens* is also nested in this clade. Bayesian binary MCMC analysis remains inconclusive with respect to the origin of this clade. There is high support for the Oceania origin for *M. prostratus*, *M. repens*, and *M. uvedaliae* (PP: 98.53%), but PP values are not decisive with respect to the origin of the whole clade (45.58%: Oceania origin, 30.35%: North America origin). Highly supported clade consisting of *M. tenellus*, *M. nepalensis*, *M. szechuanensis*, and *M. bodinieri* is found in both ML and Bayesian analyses (BS: 92%, PP: 1.0). Bayesian Binary MCMC analysis of biogeographic origin strongly suggests an Asian origin for this clade (PP: 93.58%). Another species with Asian distribution, *M. sessiflorus*, is not clustered with the rest of the Asian taxa in either ML or Bayesian analysis, and the position of this species is poorly resolved in both trees. *M. depressus*, *M. cupreus*, and *M. luteus* form a strongly supported clade in both ML and Bayesian analyses (BS: 97%, PP: 1.0). For this clade, a South American origin was suggested by Bayesian MCMC analysis of biogeographic origin (PP: 82.67%). An alternative to a South American origin, the clade might also have originated in the Americas (North and South America together), but the PP values are too weak to support this view (PP: 13.40%).

Both MP and ML analysis showed that the ancestor of the genus *Mimulus* was unlikely to have reddish flowers. Likelihood values for 1 (reddish) is 0.02 and for 0 (not reddish) is 0.98. Out of 95 *Mimulus* species, 14 of them have RO flowers. There are 2 major clades with RO flowers: *M. bifidus*, *M. flemingii*, *M. aurantiacus*, *M. longiflorus*, *M. puniceus* (RO), and *M. clevelandii *(not RO) form one of the red clades, with a potential RO-flowered common ancestor (1: 0.622, 0: 0.378). The other clade with a potential RO-flowered ancestor includes the species *M. rupestris*, *M. cardinalis*, *M. verbenaceus*, *M. eastwoodiae*, and *M. nelsonii* (RO) and *M. lewisii*, and *M. parishii* (not RO) (1: 0.628, 0: 0.372). According to our ancestral reconstruction analysis, all the RO flowered *Mimulus* taxa have appeared later than 20 Ma, which is intriguingly consistent with the approximate time for the hummingbird origins in the Americas [45].

### 3.4. Phylogenetic Comparative Analysis

#### 3.4.1. Differential Diversification with Differing Floral Colours

We find that specialization, measured narrowly as having the trait state of “reddish” flowers, is not associated with higher speciation rates (Table 1; *P* = 0.196). However, the rate of transition to the trait state of “reddish flowers” (i.e., the ability to evolve red flowers) is different from the rate at which a lineage transitions away from “reddish flowers” (Table 1; *P* = 0.02895); that is, losing red coloration happens far more frequently than gaining it. Finally, we could not find any evidence suggesting that clades with “reddish” flowers experience differential extinction rates than those without “reddish” flowers (*P* = 0.7996).

#### 3.4.2. Differential Diversification at Different Elevations

**Figure 2:**
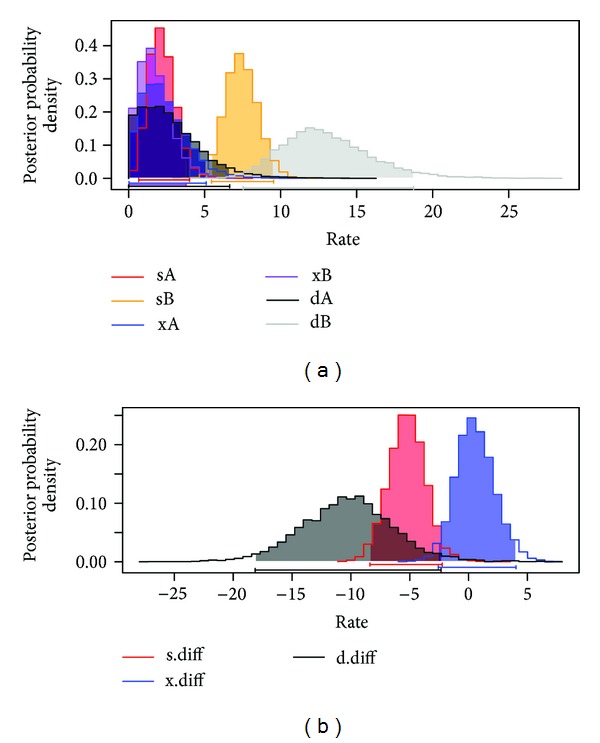
Posterior probability distributions for the six-rate GeoSSE model for the tree shown in Figure 1. (a) The parameters are speciation within low elevation regions (sA), speciation within high elevation regions (sB), and between-region speciation. (sAB), extinction from low elevation regions (xA) extinction from high elevation regions. (xB), dispersal from A to B (range expansion, dA) and dispersal from B to A (dB). Differences in dispersal are greater for range expansion from B (high elevation) to A (b) yet higher speciation rates in high elevation environments can still be observed.

Parameters from our GeoSSE analysis are provided in Table 1. In *Mimulus*, we can reject the model of equal speciation and extinction at different elevational bands, concluding that high elevation regions experience increases in diversification (Figure 2; *P* = 0.002). With this being an intriguing, yet somewhat surprising result, we reran the analyses with a small subset of bootstrap trees and found the result to be robust to phylogenetic uncertainty (*P* values ranging from 0.002 to <0.0001, *N* = 10). Including the between-region mode of speciation does not, however, significantly improve the fit (*P* = 1.00). We also find that high elevation lineages experience increased range expansion into lower elevations more so than the reverse (Figure 2; *P* = 0.02).

#### 3.4.3. Correlated Transitions between Red Flowers and High Elevations

We provide maximum likelihood parameters from our MuSSE analysis in Table 1. Our MuSSE analysis tested whether certain transitions were more common than others (e.g., we can test if RO flowers evolve more often in high elevation environments), yet we did not find any evidence that these transition rates were unequal (*P* = 0.21) in such a way that would result in a disproportionate number of RO-flowered species in high elevation locations (see Figure 1).

## 4. Discussion

We hypothesized that red flowered lineages might be restricted to high elevation environments as others have posited [15] and that this might contribute to lower speciation rates. Surprisingly, we found no indication that red flowered species are more prevalent at high elevation, yet high elevation bands were actually regions of increased speciation. In addition, we found no indication that speciation accompanied shifts between elevational zones. Previous studies have also found that diversification rates increase in mountainous regions [10, 46] and our study in *Mimulus* corroborates these findings.

Finally, the species that originate in high elevation environments show an increased propensity to experience range expansion into low elevation environments, supporting other studies that suggest that the increased diversification of alpine regions provides a “species pump” for global species richness [46, 47].

Floral colour can be considered as a secondary sexual characteristic in flowering plants, somewhat analogous to exaggerated ornaments in animals [48]. Because floral colour provides an important cue for pollinators, differences in colour can translate to attracting an increased number of visits from efficient pollinators, which often results in increased pollen transfer [49] and a reduction in the amount of inbreeding [50]. Reddish flowers are typically associated with pollination by birds [51], though this relationship is far from absolute. In *Mimulus*, a primarily North American lineage, bird pollination is almost exclusively hummingbird pollination. While hummingbird pollination is thought to be an extremely efficient means of dispersing pollen and evolving reddish flowers may be an adaptation for bird pollination, we did not find that the evolution or red/orange flowers was associated with increased speciation rates or decreased extinction rates, and the character state appears to be more ephemeral than a non-RO phenotype. Also, RO lineages were not restricted to high elevation environments as has been supposed [15], yet we find that high elevation environments were hotspots for speciation, possibly due to the topographical heterogeneity presented by isolated mountain peaks [10].

Potential reasons for the increased transition rates away from the RO phenotype involve scenarios that incorporate the ease at which a mutant can arise and the probability that such a mutation can fix. One scenario that might be included is that the flowers themselves are costly (red-hued flowers are associated with hummingbird pollination and these flowers might also be large and nectar rich), and so the phenotype is only selected in environments where other pollinators (e.g., bees) are scarce. Furthermore, there are many nonpollinator agents of selection as well as genetic arguments that may be responsible for this pattern and the ease of transitions away from an RO phenotype is not devoid of empirical evidence. Yellow morphs have been observed to be easily established in more than one population of *Mimulus cardinalis* [52]. While transitions from blue to red have been observed to be more common than the reverse [16], there has been little work done on the transitions between red and yellow flowers (the most common alternate floral colour in *Mimulus*). Transitions from pigmented to nonpigmented flowers are thought to be more common than the reverse generally [16], and the agents of selection may or may not include pollinators [53]. Phylogenetic comparisons in *Ipomoea* revealed that transition rates between the two character states were roughly equal but nonpigmented flowers had lower speciation rates [54]. White flowers are potentially an adaptation for moth pollination [55], yet might also be selectively favoured if the resources for costly pigments can be coopted for other functions. Because *Mimulus* includes few nonpigmented (white) flowered species, direct comparisons with previous studies are difficult because yellow flowers still contain flavonol pigments.

Another potential explanation to why transitions to red flowers are rare in *Mimulus* could be that the hummingbird family (a potential selective agent) originated about 20 mya [45], which is relatively recent when compared to the history of *Mimulus*. In other words, the ecological opportunity for adapting to the hummingbird pollination syndrome was not a possible transition for much of the tree space we analyzed in *Mimulus* [56]. Further studies on pollination syndromes that examine transition rates (and speciation rates) before and after the origins of hummingbirds with direct observations of pollinator visitation rates might be able to refine the role that the origin of a new mutualist has had on North American angiosperm genera.

## Supplementary Material

Here, we provide the three gene Maximum Likelihood (SuppFig. 1) and Bayesian (SuppFig 2) phylogenies. We also provide the results of ancestral range reconstruction analysis on Mimulus using Bayesian Binary MCMC (SuppFig3).

## Figures and Tables

**Table 1:** Results of BiSSE, GeoSSE, and MuSSE analyses of RO phenotype and high elevation in *Mimulus.* In the BiSSE/GeoSSE analysis, we use the notation of non-RO = 0 and RO = 1, low elevation = 0, and high elevation = 1 (and the GeoSSE analysis allows for third category for species having ranges that span low and high elevation). In the MuSSE analysis, trait combinations use the following notation: 1: low elevation, no-RO; 2: low elevation, RO; 3: high elevation, no-RO; 4: high elevation, RO. We test whether the evolution of the RO phenotype depends on the background of elevation (does 1 → 2 differ from 3 → 4 or 1 → 3 from 2 → 4, etc.). *P < 0.05 and **  <  0.001.

Model	lnLik	AIC
*Differential diversification of red-flowered lineages *		
Full model (*λ*_0_ = 6.88; *λ*_1_ = 12.76; *μ*_0_ = 0; *μ*_1_ = 3.3; *q*_01_ = 0.42; *q*_10_ = 5.36)	70.21	−128.42
*λ*_1_ = *λ*_0_	69.37	−128.75
*μ*_1_ = *μ*_0_	70.18	−130.35
*q*_10_ = *q*_01_	67.82*	−125.65
*Differential diversification of high elevation lineages *		
Full model (*λ*_0_ = 0.72; *λ*_1_ = 9.69; *λ*_01_ = 0; *μ*_0_ = 16.13; *μ*_1_ = 5.08; d_01_ = 0; d_10_ = 45.46)	−10.05	34.09
*λ*_01_ = 0 (between-region mode of speciation)	−10.05	32.09
*λ*_1_ = *λ*_0_	−16.19**	42.37
d_10_ = d_01_	−13.22*	38.44
*Correlated evolution of red flowers and high elevation *		
Full model (*λ* = 7.20; *μ* = 0.00; *q*_12_ = 0.101; *q*_34_ = 0.997; *q*_21_ = 2.301; *q*_43_ = 7.208; *q*_13_ = 8.157; *q*_24_ = −8.157; *q*_31_ = 2.350; *q*_42_ = −2.179)	14.74	
*q*_12_ = *q*_34_, *q*_13_ = *q*_24_, *q*_21_ = *q*_43_, *q*_31_ = *q*_42_	17.51	

**Table 2:** Elevation ranges, geographical distribution, and qualitative assessments of floral colour, as well as Genbank accession numbers for the species of *Mimulus* used in this study.

Species	Color	Dist.	Elev. (m)	ETS	ITS	trnL-F
*Mimulus alsinoides *	Yellow	N. America	0–860	AY575340.1	AY575450.1	AY575542.1
*Mimulus ampliatus *	Yellow	N. America	610–1067	AY575310.1	AY575421.1	AY575517.1
*Mimulus androsaceus *	Purple	N. America	0–1219	—	AY575400.1	AY575502.1
*Mimulus angustatus *	White, yellow, and pink/purple	N. America	0–1219	AY575269.1	AY575378.1	AY575485.1
*Mimulus aurantiacus *	Red/orange	N. America	0–2286	AF478950.1	AF478917.1	AF478982.1
*Mimulus bicolor *	Yellow and white	N. America	0–1829	AY575298.1	AY575409.1	AF478995.1
*Mimulus bifidus *	White/orange/yellow	N. America	0–1524	AY575283.1	AY575391.1	AY575496.1
*Mimulus bigelovii *	Pink/purple	N. America	120–2300	AY575242.1	AY575353.1	AY575461.1
*Mimulus bodinieri *	—	Asia	—	AY575323.1	AY575434.1	—
*Mimulus bolanderi *	Pink/purple	N. America	0–1981	AY575241.1	AY575352.1	AY575460.1
*Mimulus breviflorus *	Yellow	N. America	1450–2200	AY575314.1	AY575425.1	AY575521.1
*Mimulus brevipes *	Yellow	N. America	1524–2164	AY575247.1	AY575358.1	AY575466.1
*Mimulus breweri *	Pink/purple	N. America	1219–3353	—	—	AY575509.1
*Mimulus cardinalis *	Red	N. America	0–2438	AF478964.1	AY575414.1	AF478996.1
*Mimulus clevelandii *	Yellow	N. America	915–1465	AY575278.1	AY575386.1	AF478983.1
*Mimulus clivicola *	Pink	N. America	488–1250	AY575258.1	AY575369.1	AY575477.1
*Mimulus congdonii *	Pink/purple	N. America	0–914	AY575275.1	AY575383.1	AY575489.1
*Mimulus constrictus *	Pink/purple	N. America	750–2200	AY575238.1	AY575349.1	AY575457.1
*Mimulus cupreus *	Red/orange	S. America	2300	AY575336.1	AY575447.1	AY575540.1
*Mimulus cusickii *	Pink/purple	N. America	600–1600	AY575256.1	AY575367.1	AY575475.1
*Mimulus dentatus *	Yellow	N. America	0–397	AY575338.1	AY575449.1	—
*Mimulus dentilobus *	Yellow	N. America	—	AY575333.1	AY575444.1	AY575537.1
*Mimulus depressus *	Yellow	S. America	0–1158	AF478961.1	AY575446.1	AY575539.1
*Mimulus douglasii *	Pink/purple	N. America	0–1219	AY575273.1	AY575381.1	AF478984.1
*Mimulus dudleyi *	Yellow	N. America	Lower elevations	AY575321.1	AY575432.1	AY575528.1
*Mimulus eastwoodiae *	Red	N. America	1433–1768	AY575306.1	AY575417.1	—
*Mimulus evanescens *	Yellow	N. America	1250–1700	AY575347.1	AY575455.1	AY575547.1
*Mimulus exiguus *	Pink/purple	N. America	1640	AY575309.1	AY575420.1	AY575516.1
*Mimulus filicaulis *	Pink/purple	N. America	1219–1524	AY575299.1	AY575410.1	AY575511.1
*Mimulus flemingii *	Red/orange	N. America	—	AY575287.1	AY575395.1	AY575499.1
*Mimulus floribundus *	Yellow	N. America	0–3353	AF478959.1	AF478926.1	AF478991.1
*Mimulus fremontii *	Pink/purple	N. America	75–2100	AY575246.1	AY575357.1	AY575465.1
*Mimulus gemmiparus *	Yellow	N. America	Higher elevations	AY575341.1	AY575451.1	AY575543.1
*Mimulus glabratus *	Yellow	N. America	1800–3200	AY575334.1	AY575445.1	AY575538.1
*Mimulus glaucescens *	Yellow	N. America	0–762	AY575332.1	AY575443.1	AY575536.1
*Mimulus gracilipes *	Pink/purple	N. America	—	AY575296.1	AY575407.1	AY575508.1
*Mimulus gracilis *	Purple	Oceania	250–1530	AY575345.1	AF478934.1	AF478999.1
*Mimulus guttatus *	Yellow	N. America	0–3048	AY575328.1	AY575439.1	AY575533.1
*Mimulus hymenophyllus *	Yellow	N. America	1086	AY575311.1	AY575422.1	AY575518.1
*Mimulus inconspicuus *	Pink/purple	N. America	0–2896	AY575342.1	AY575452.1	AY575544.1
*Mimulus jepsonii *	Pink/purple	N. America	1219–2743	AY575259.1	AY575370.1	AY575478.1
*Mimulus johnstonii *	Pink/purple	N. America	1219–2134	AY575248.1	AY575359.1	AY575467.1
*Mimulus jungermannioides *	Yellow	N. America	152–1006	AY575315.1	AY575426.1	AY575522.1
*Mimulus kelloggii *	Pink/purple	N. America	0–914	AY575274.1	AY575382.1	AY575488.1
*Mimulus latidens *	Light pink	N. America	0–762	AY575318.1	AY575429.1	AY575525.1
*Mimulus layneae *	Pink	N. America	0–2286	AY575260.1	AY575371.1	AY575479.1
*Mimulus leptaleus *	Pink/purple	N. America	1950–3353	AY575239.1	AY575350.1	AY575458.1
*Mimulus lewisii *	Pink	N. America	1219–3048	AF478965.1	AF478932.1	AF478997.1
*Mimulus longiflorus *	Red/orange	N. America	0–2286	AY575285.1	AY575393.1	AY575498.1
*Mimulus luteus *	Yellow	S. America	1400–2900	AY575337.1	AY575448.1	AY575541.1
*Mimulus mephiticus *	Pink	N. America	1219–3658	AY575244.1	AY575355.1	AY575463.1
*Mimulus micranthus *	Yellow	N. America		AY575329.1	AY575440.1	AY575534.1
*Mimulus mohavensis *	White and pink	N. America	600–1200	AY575264.1	AY575375.1	AY575482.1
*Mimulus montioides *	Yellow	N. America	914–5553	AY575294.1	AY575405.1	AY575506.1
*Mimulus moschatus *	Yellow	N. America	0–2682	AY575317.1	AY575428.1	AY575524.1
*Mimulus nanus *	Pink/purple	N. America	1372–2286	AY575249.1	AY575360.1	AY575468.1
*Mimulus nelsonii *	Red	N. America	2438–2743	AY575302.1	AY575413.1	—
*Mimulus nepalensis *	Yellow	Asia	0–867	AY575324.1	AY575435.1	AY575530.1
*Mimulus norrisi *	Yellow	N. America	—	AY575322.1	AY575433.1	AY575529.1
*Mimulus nudatus *	Yellow	N. America	250–700	AY575330.1	AY575441.1	AY575535.1
*Mimulus palmeri *	Pink	N. America	0–2134	AY575295.1	AY575406.1	AY575507.1
*Mimulus parishii *	Light pink	N. America	0–2100	AY575300.1	AY575411.1	AY575512.1
*Mimulus parryi *	Pink/purple	N. America	1200–2600	AY575277.1	AY575385.1	AY575491.1
*Mimulus patulus *	Yellow	N. America	305–610	AY575312.1	AY575423.1	AY575519.1
*Mimulus pictus *	Purple and white	N. America	305–1219	AY575265.1	AY575376.1	AY575483.1
*Mimulus pilosus *	Yellow	N. America	0–2591	AY575266.1	—	—
*Mimulus primuloides *	Yellow	N. America	600–3414	AY575308.1	AY575419.1	AY575515.1
*Mimulus prostratus *	Purple and white	Oceania	—	AY943099.1	AY943125.1	AY943150.1
*Mimulus pulchellus *	Yellow and pink/purple	N. America	914–1524	AF478953.1	AF478920.1	AF478985.1
*Mimulus pulsiferae *	Yellow	N. America	762–1524	AY575313.1	AY575424.1	AY575520.1
*Mimulus puniceus *	Red/orange	N. America	—	AY575279.1	AY575387.1	AY575493.1
*Mimulus purpureus *	Purple	N. America	Lower elevations	—	AY575402.1	AY575504.1
*Mimulus pygmaeus *	Yellow	N. America	500–1840	AY575272.1	—	—
*Mimulus rattanii *	Pink/purple	N. America	260	AY575245.1	AY575356.1	AY575464.1
*Mimulus repens *	Purple	Oceania	45	AY943088.1	AY943115.1	—
*Mimulus ringens *	Purple	N. America	0–200	AY575344.1	AY575454.1	AF479000.1
*Mimulus rubellus *	Pink	N. America	800–3600	AY575297.1	AY575408.1	AY575510.1
*Mimulus rupestris *	Red	N. America	2286	AY575301.1	AY575412.1	AY575513.1
*Mimulus rupicola *	Light pink/purple	N. America	305–1798	AY575276.1	AY575384.1	AY575490.1
*Mimulus sessilifolius *	Yellow	Asia	0–2707	AY575339.1	—	—
*Mimulus shevockii *	Yellow	N. America	823–1341	—	AY575403.1	AY575505.1
*Mimulus suksdorfii *	Yellow	N. America	1524–3962	AY575292.1	AY575401.1	AY575503.1
*Mimulus szechuanensis *	Yellow	Asla	—	—	FJ172743.1	FJ172692.1
*Mimulus tenellus *	Yellow	Asia	0–3048	AY575325.1	AY575436.1	FJ172691.1
*Mimulus tilingii *	Yellow	N. America	1950–3658	AY575330.1	AY575442.1	AF478994.1
*Mimulus torreyi *	Pink/purple	N. America	0–2438	AY575262.1	AY575373.1	AY575481.1
*Mimulus tricolor *	White, yellow and pink/purple	N. America	0–610	AY575268.1	AY575377.1	AY575484.1
*Mimulus uvedaliae *	Purple and white	Oceania	—	AY575346.1	AF478936.1	AF479001.1
*Mimulus verbenaceus *	Red	N. America	985–1676	AY575307.1	AY575418.1	—
*Mimulus viscidus *	White and pink/red	N. America	610–1829	AY575243.1	AY575354.1	AY575462.1
*Mimulus washingtonensis *	Yellow	N. America	—	AY575316.1	AY575427.1	AY575523.1
*Mimulus whitneyi *	Pink or yellow	N. America	1829–3353	AY575237.1	AY575348.1	AY575456.1
*Mimulus wiensii *	—	N. America	678–797	AY575326.1	AY575437.1	AY575531.1
*Mimulus yeocorensis *	—	N. America	—	AY575327.1	AY575438.1	AY575532.1
*Mohavea breviflora *				AF478979.1	AF513892.1	AF479011.1

## References

[1] Igic B, Lande R, Kohn JR (2008). Loss of self-incompatibility and its evolutionary consequences. *International Journal of Plant Sciences*.

[2] Sargent RD (2004). Floral symmetry affects speciation rates in angiosperms. *Proceedings of the Royal Society B*.

[3] Ricklefs RE (2007). History and diversity: explorations at the intersection of ecology and evolution. *American Naturalist*.

[4] Rabosky DL (2009). Ecological limits and diversification rate: alternative paradigms to explain the variation in species richness among clades and regions. *Ecology Letters*.

[5] Vamosi JC, Vamosi SM (2010). Key innovations within a geographical context in flowering plants: towards resolving Darwin’s abominable mystery. *Ecology Letters*.

[6] Vamosi SM, Vamosi JC (2012). Causes and consequences of range size variation: the influence of traits, speciation, and extinction. *Frontiers of Biogeography*.

[7] Owens IPF, Bennett PM, Harvey PH (1999). Species richness among birds: body size, life history, sexual selection or ecology?. *Proceedings of the Royal Society B*.

[8] Böhning-Gaese K, Caprano T, van Ewijk K, Veith M (2006). Range size: disentangling current traits and phylogenetic and biogeographic factors. *American Naturalist*.

[9] Losos JB, Schluter D (2000). Analysis of an evolutionary species-area relationship. *Nature*.

[10] Hughes C, Eastwood R (2006). Island radiation on a continental scale: exceptional rates of plant diversification after uplift of the Andes. *Proceedings of the National Academy of Sciences of the United States of America*.

[11] Armbruster WS (2002). Can indirect selection and genetic context contribute to trait diversification? A transition-probability study of blossom-colour evolution in two genera. *Journal of Evolutionary Biology*.

[12] Arnold SEJ, Savolainen V, Chittka L (2009). Flower colours along an alpine altitude gradient, seen through the eyes of fly and bee pollinators. *Arthropod-Plant Interactions*.

[13] van der Pijl L, Richards AJ (1978). Reproductive integration and sexual disharmony in floral functions. *The Pollination of Flowers By Insects*.

[14] Ollerton J, Alarcón R, Waser NM (2009). A global test of the pollination syndrome hypothesis. *Annals of Botany*.

[15] Cruden RW (1972). Pollinators in high-elevation ecosystems: relative effectiveness of birds and bees. *Science*.

[16] Rausher MD (2008). Evolutionary transitions in floral color. *International Journal of Plant Sciences*.

[17] Grossenbacher DL, Whittall JB (2011). Increased floral divergence in sympatric monkeyflowers. *Evolution*.

[18] Beardsley PM, Schoenig SE, Whittall JB, Olmstead RG (2004). Patterns of evolution in western North American Mimulus (Phrymaceae). *American Journal of Botany*.

[19] Whittall JB, Carlson ML, Beardsley PM, Meinke RJ, Liston A (2006). The Mimulus moschatus alliance (Phrymaceae): molecular and morphological phylogenetics and their conservation implications. *Systematic Botany*.

[20] Beardsley PM, Olmstead RG (2002). Redefining phrymaceae: the placement of *Mimulus*, tribe mimuleae, and *Phryma*. *American Journal of Botany*.

[21] California Native Plant Society (CNPS) Inventory of Rare and Endangered Plants.

[22] Calflora Information on California plants for education, research and conservation.

[23] EOL Encyclopedia of Life. http://eol.org/.

[24] Project JF http://ucjeps.berkeley.edu/IJM.html.

[25] FloraBase FloraBase—the Western Australian Flora. http://florabase.dpaw.wa.gov.au/.

[26] USDA, NRCS http://plants.usda.gov/java/.

[27] Olmstead RG, Depamphilis CW, Wolfe AD, Young ND, Elisons WJ, Reeves PA (2001). Disintegration of the scrophulariaceae. *American Journal of Botany*.

[28] Maddison WP, Maddison DR Mesquite: a modular system for evolutionary analysis. http://mesquiteproject.org/mesquite/mesquite.html.

[29] Wheeler TJ, Kececioglu JD (2007). Multiple alignment by aligning alignments. *Bioinformatics*.

[30] Stamatakis A (2006). RAxML-VI-HPC: maximum likelihood-based phylogenetic analyses with thousands of taxa and mixed models. *Bioinformatics*.

[31] Drummond AJ, Rambaut A (2007). BEAST: bayesian evolutionary analysis by sampling trees. *BMC Evolutionary Biology*.

[32] Drummond AJ, Suchard MA, Xie D, Rambaut A (2012). Bayesian phylogenetics with BEAUti and the BEAST 1.7. *Molecular Biology and Evolution*.

[33] Hasegawa M, Kishino H, Yano T (1985). Dating of the human-ape splitting by a molecular clock of mitochondrial DNA. *Journal of Molecular Evolution*.

[34] Bremer K, Friis EM, Bremer B (2004). Molecular phylogenetic dating of asterid flowering plants shows early cretaceous diversification. *Systematic Biology*.

[35] Gernhard T (2008). The conditioned reconstructed process. *Journal of Theoretical Biology*.

[36] Yule GU (1925). A mathematical theory of evolution based on the conclusions of Dr. J.C. Willis. *Philosophical Transactions of the Royal Society B*.

[37] Maddison WP, Midford PE, Otto SP (2007). Estimating a binary character’s effect on speciation and extinction. *Systematic Biology*.

[38] Fitzjohn RG Diversitree: comparative phylogenetic tests of diversification.

[39] R Development Core Team R: A language and environment for statistical computing.

[40] Yu Y, Harris AJ, He XJ (2010). S-DIVA (Statistical Dispersal-Vicariance Analysis): a tool for inferring biogeographic histories. *Molecular Phylogenetics and Evolution*.

[41] Ronquist F (1997). Dispersal-vicariance analysis: a new approach to the quantification of historical biogeography. *Systematic Biology*.

[42] Ronquist F (2004). Bayesian inference of character evolution. *Trends in Ecology and Evolution*.

[43] Ree RH, Smith SA (2008). Maximum likelihood inference of geographic range evolution by dispersal, local extinction, and cladogenesis. *Systematic Biology*.

[44] Beardsley PM, Yen A, Olmstead RG (2003). AFLP phylogeny of *Mimulus* section *Erythranthe* and the evolution of hummingbird pollination. *Evolution*.

[45] Bleiweiss R (1998). Origin of hummingbird faunas. *Biological Journal of the Linnean Society*.

[46] Schoville SD, Roderick GK, Kavanaugh DH (2012). Testing the “Pleistocene species pump” in alpine habitats: lineage diversification of flightless ground beetles (Coleoptera: Carabidae: Nebria) in relation to altitudinal zonation. *Biological Journal of the Linnean Society*.

[47] Sedano RE, Burns KJ (2010). Are the Northern Andes a species pump for Neotropical birds? Phylogenetics and biogeography of a clade of Neotropical tanagers (Aves: Thraupini). *Journal of Biogeography*.

[48] Biernaskie JM, Elle E (2007). A theory for exaggerated secondary sexual traits in animal-pollinated plants. *Evolutionary Ecology*.

[49] Engel EC, Irwin RE (2003). Linking pollinator visitation rate and pollen receipt. *American Journal of Botany*.

[50] Bosch M, Waser NM (1999). Effects of local density on pollination and reproduction in *Delphinium nuttallianum* and *Aconitum columbianum* (Ranunculaceae). *American Journal of Botany*.

[51] Rodríguez-Gironés MA, Santamaría L (2004). Why are so many bird flowers red?. *PLoS Biology*.

[52] Vickery RK (1995). Speciation in *Mimulus*, or, can a simple flower color mutant lead to species divergence?. *Great Basin Naturalist*.

[53] Strauss SY, Irwin RE, Lambrix VM (2004). Optimal defence theory and flower petal colour predict variation in the secondary chemistry of wild radish. *Journal of Ecology*.

[54] Smith SD, Miller RE, Otto SP, FitzJohn RG, Rausher MD, Long M, Gu H, Zhou Z, editors (2010). The effects of flower color transitions on diversification rates in morning glories (Ipomoea subg. Quamoclit, Convolvulaceae). *Darwin's Heritage Today*.

[55] Smith SD (2010). Using phylogenetics to detect pollinator-mediated floral evolution. *New Phytologist*.

[56] Rabosky DL (2009). Ecological limits on clade diversification in higher taxa. *American Naturalist*.

