# Supplementary material for: Diversification in Monkeyflowers: An Investigation of the Effects of Elevation and Floral Color in the Genus Mimulus
Source: Int J Evol Biol. 2014 Jan 5;2014:382453. doi: 10.1155/2014/382453 (PMC3913381; doi:10.1155/2014/382453)

SuppFigure 1. Phylogenetic tree for Mimulus using Maximum Likelihood method with all 3 markers we have data for. Node labels= bootstrap percentages

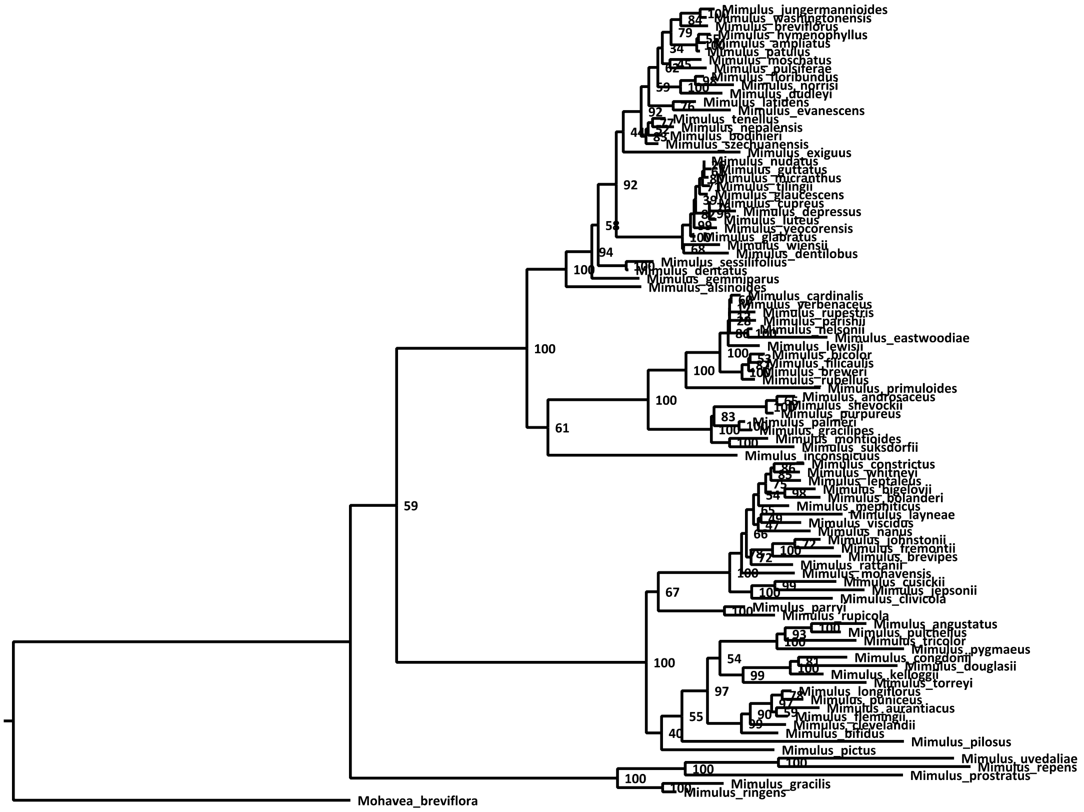

SupplFig2. Phylogenetic tree for Mimulus using Bayesian method with all 3 markers we have data for. Node labels= age, branch labels = posterior with colour coding (the values from 0 to 1 is shown as a colour gradient from red to green).

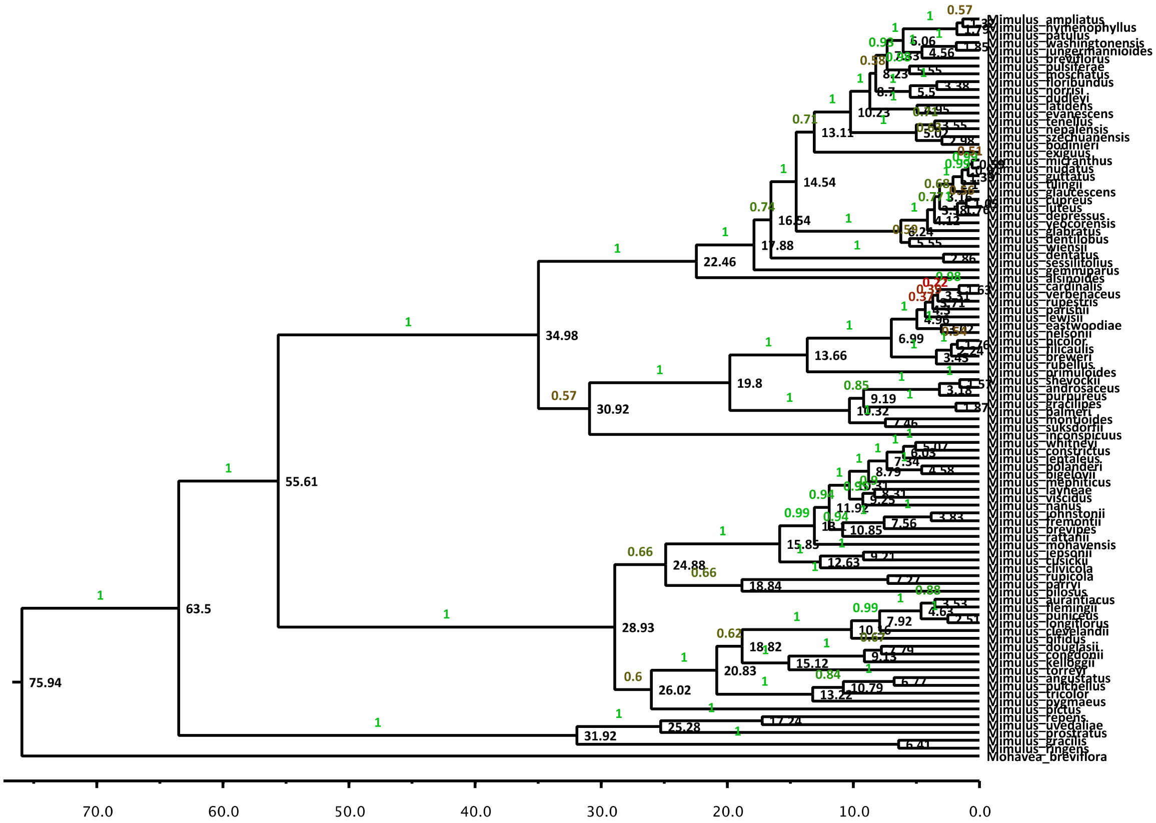

SuppFig3. Bayesian Binary MCMC Distribution Tree: Ancestral Distribution of Mimulus on the Bayesian Tree. A= North America (blue), B= South America (Purple), C= Asia (Yellow), D= Oceania (Red).

result of combined:

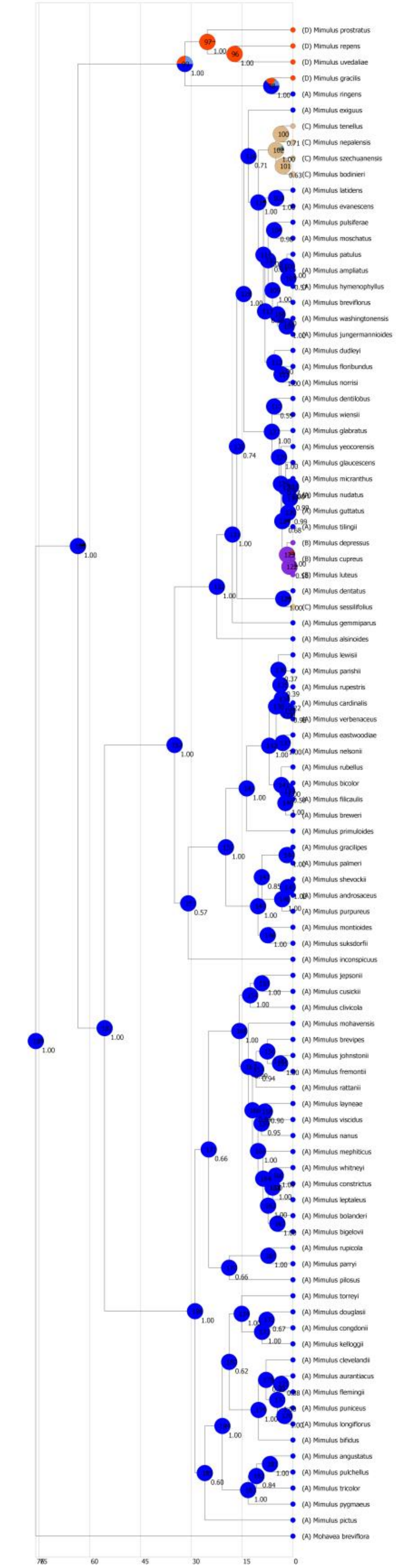

Supplement: Supplementary file 1 — Here, we provide the three gene Maximum Likelihood (SuppFig. 1) and Bayesian (SuppFig 2) phylogenies. We also provide the results of ancestral range reconstruction analysis on Mimulus using Bayesian Binary MCMC (SuppFig3). [file 382453.f1.pdf]
